# Multi-parameter adjustment for high-precision azimuthal intersection positioning

**DOI:** 10.1016/j.mex.2020.100968

**Published:** 2020-06-20

**Authors:** Songtao Ai, Shansi Wang, Yuansheng Li, Leibao Liu

**Affiliations:** a Chinese Antarctic Center of Surveying & Mapping, Wuhan University, Wuhan 430079, China; b Polar Research Institute of China, Shanghai 20036, China

**Keywords:** Space intersection, Parameter adjustment model, Earth curvature error correction, Atmospheric error correction

## Abstract

The traditional azimuthal intersection method is viable for situations with only two control stations but a simple height averaging is not rigorous because the intersections vary in their distances from the two stations. In order to obtain the high-precision azimuthal intersections, this study presented a multi-parameter adjustment method, together with the Earth curvature correction and the atmospheric refraction correction models. This method is robust with varied distances between the control stations and the targeted intersections, without limitation of station quantity.

• *Based on the traditional space intersection, a multi-parameter adjustment model is added into the data processing for high-precision 3D positioning.*
• *Both the Earth curvature error correction model and the atmospheric error correction model are included in the multi-parameter adjustment model, so the intersected points are more accurate than traditional intersections.*

## Graphical abstract

**Figure:**
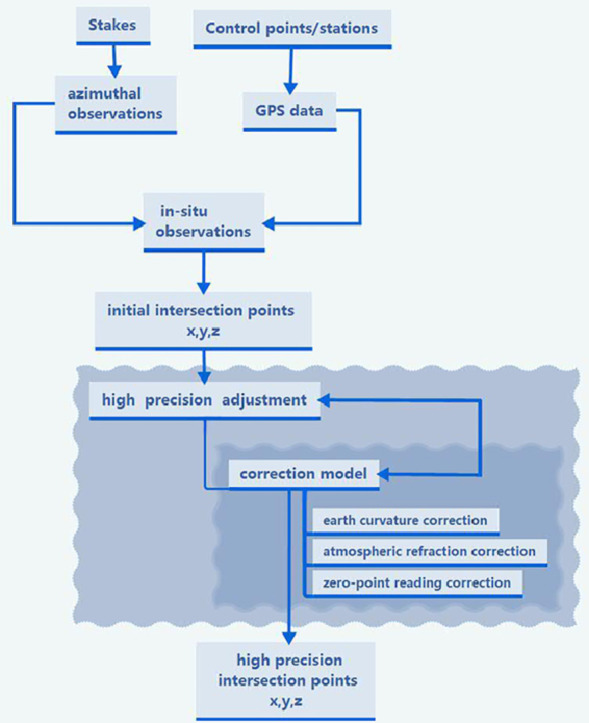

**Specifications Table**

**Table:** 

Subject Area:	• **Earth and Planetary Sciences**
More specific subject area:	*Surveying and Positioning*
Method name:	**high-precision azimuthal intersection positioning**
Name and reference of original method:	
Resource availability:	

## Method details

### Azimuthal intersection method

In order to get the position of a target far away from the observatory, space intersection method is a good choice. All the azimuthal data can be collected by total stations or theodolites on the control stations. Using the classic space azimuthal intersection method, at least two control stations are needed to obtain the 3D coordinates of the target.

**Fig. 1:**
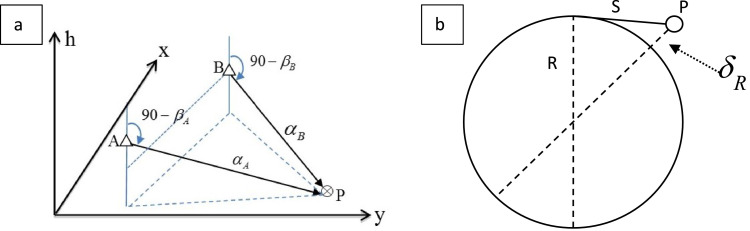
a Sketch of the spatial azimuthal intersection method. A is one control Station, B is another control station and P is a targeted intersection such as a stake on the glacier. b Sketch of the earth curvature correction, the value of R is 6,378,137 m and the geodetic system is WGS84.

When two control stations, named A and B, are used to position one target, there are two pairs of horizontal and vertical azimuths from the stations at A and B. From these measurements, the 3D location of a stake can be calculated, and one redundant vertical azimuth exists, as shown in Fig. 1a. Due to errors in the observations, the heights calculated from the vertical azimuths at both A and B differed, i.e., there was a vertical bias inside one intersection. Given that the locations of station A and station B were (*x*_A_*, y*_A_*, h*_A_) and(*x*_B_*, y*_B_*, h*_B_), respectively, as acquired independently via GPS, and that the observed azimuthal records were (*α*_A_*,β*_A_) and (*α*_B_*,β*_B_) from stations A and B, respectively, the 3D location of a stake was traditionally calculated using Eq. (1) [5].

(1) $$\begin{array}{c} x=\left(x_{A}tan\alpha_{A}-x_{B}tan\alpha_{B}+y_{B}-y_{A}\right)/\left(tan\alpha_{A}-tan\alpha_{B}\right)\\ y=\left(y_{A}ctn\alpha_{A}-y_{B}ctn\alpha_{B}+x_{B}-x_{A}\right)/\left(ctn\alpha_{A}-ctn\alpha_{B}\right)\\ h=(h_{A}+S_{A}tan\beta_{A}+h_{B}+S_{B}tan\beta_{B})/2 \end{array}\}$$

In Eq. (1), *S*_A_ and *S*_B_ are the horizontal distances from the intersection point P and the stations, $S_{i}=\sqrt{{\left(x-x_{i}\right)}^{2}+{\left(y-y_{i}\right)}^{2}}$ and *i* ∈ {A, B}.

The traditional space intersection method, as shown in Eq. (1), is viable for situations with only two stations. Moreover, the *S_i_* varied from the stations. Therefore, a simple height averaging is not suitable for the error distributions at different distances, which means that the height averaging method is not rigorous.

In order to obtain high-precision location of the intersection point P(*x*_P_, *y*_P_, *h*_P_), a multi-parameter adjustment model can be used to obtain the best solution. In this process, iterative calculations should be used, and the desired coordinates of the intersection point are set as the unknown parameters.

Based on the traditional space intersection, we used Eq. (1) to calculate the approximate intersected coordinates (*x, y, h*), and then to calculate the approximate horizontal and vertical azimuths via Eq. (2).

(2) $$\begin{array}{c} \alpha_{i}=\arccos\frac{x-x_{i}}{S_{i}}\\ \beta_{i}=\arctan\frac{h-h_{i}}{S_{i}} \end{array}\}$$

Here, the horizontal azimuth has a scope of 0 ≤ *α* < 2*π*, and the vertical azimuth has a scope of −π/2<β<π/2. Note that the value of *α* in Eq. (2) should be decided by coordinate y. When $y-y_{i}<0$, $\alpha =2\pi -\alpha_{i}$. Suppose the corrections of the coordinates of P are$\left(\hat{x},\hat{y},\hat{h}\right)$, then the corrections of the azimuths, $\delta_{\alpha_{i}}$ and $\delta_{\beta_{i}}$, are as shown in Eq. (3) from a Taylor series expansion:

(3) $$\begin{array}{c} \delta_{\alpha_{i}}=-\frac{y-y_{i}}{{S_{i}}^{2}}\hat{x}+\frac{x-x_{i}}{{S_{i}}^{2}}\hat{y}\\ \delta_{\beta_{i}}=-\frac{\left(x-x_{i}\right)\left(h-h_{i}\right)}{S_{i}\left({S_{i}}^{2}+{\left(h-h_{i}\right)}^{2}\right)}\hat{x}-\frac{\left(y-y_{i}\right)\left(h-h_{i}\right)}{S_{i}\left({S_{i}}^{2}+{\left(h-h_{i}\right)}^{2}\right)}\hat{y}+\frac{S_{i}}{{S_{i}}^{2}+{\left(h-h_{i}\right)}^{2}}\hat{h} \end{array}\}$$

Eq. (3) is the linear relationship between the coordinate corrections and the azimuthal corrections, which can be used for the programming, with consideration of the identical unit.

### The parameter adjustment model

Since the errors in this study are mainly caused by the azimuthal observations, we can use the parameter adjustment model [2], [3], [4] to get the best solution, as shown in Eq. (4):
  (4) $$\begin{array}{c} V=BX-L\\ V^{T}PV=\min\\ X={\left(B^{T}PB\right)}^{-1}B^{T}PL \end{array}\}$$

Here, the azimuthal correction vector $V={\left\{\delta_{\alpha_{A}},\delta_{\beta_{A}},\delta_{\alpha_{B}},\delta_{\beta_{B}}\right\}}^{T}$, the coordinate correction vector $X={\left\{\hat{x},\hat{y},\hat{h}\right\}}^{T}$, and the coefficient matrix *B* is

(5) $$B=\left\{\begin{array}{ccc} -\frac{y-y_{A}}{{S_{A}}^{2}} & \frac{x-x_{A}}{{S_{A}}^{2}} & 0\\ -\frac{\left(x-x_{A}\right)\left(h-h_{A}\right)}{S_{A}\left({S_{A}}^{2}+{\left(h-h_{A}\right)}^{2}\right)} & -\frac{\left(y-y_{A}\right)\left(h-h_{A}\right)}{S_{A}\left({S_{A}}^{2}+{\left(h-h_{A}\right)}^{2}\right)} & \frac{S_{A}}{{S_{A}}^{2}+{\left(h-h_{A}\right)}^{2}}\\ -\frac{y-y_{B}}{{S_{B}}^{2}} & \frac{x-x_{B}}{{S_{B}}^{2}} & 0\\ -\frac{\left(x-x_{B}\right)\left(h-h_{B}\right)}{S_{B}\left({S_{B}}^{2}+{\left(h-h_{B}\right)}^{2}\right)} & -\frac{\left(y-y_{B}\right)\left(h-h_{B}\right)}{S_{B}\left({S_{B}}^{2}+{\left(h-h_{B}\right)}^{2}\right)} & \frac{S_{B}}{{S_{B}}^{2}+{\left(h-h_{B}\right)}^{2}} \end{array}\right\}$$

In this study, the precisions of the horizontal and vertical observations were equal. Therefore, the weight matrix P was used as the unit matrix. The superscript T inside all the equations indicates the transposition of a vector or matrix. For example, the *V*^T^ is the transposition of vector *V*, and the *B*^T^ is the transposed matrix of *B*.

The residual vector of the azimuthal observations is $L={\left\{\alpha_{A}-{\alpha_{A}}^{0},\beta_{A}-{\beta_{A}}^{0},\alpha_{B}-{\alpha_{B}}^{0},\beta_{B}-{\beta_{B}}^{0}\right\}}^{T}$. Here, the approximate values of the azimuths (*α*_A_^0^, *β*_A_^0^, *α*_B_^0^, *β*_B_^0^) can be calculated from Eq. (2). Note that the calculation in Eq. (4) requires iteration, which means the vectors B, L, and X should be revised every time. Consequently, the calculated values of the azimuths (*α*_A_^0^, *β*_A_^0^, *α*_B_^0^, *β*_B_^0^) should also be renewed with latest (*x, y, h*) after each iteration. Actually, when all the coordinate corrections are less than a predefined threshold value (e.g., 0.001 m), the iterations should be terminated, and the adjusted coordinates can be outputted.

### Error corrections

The adjustment above reduces only the potential errors of the azimuthal observations. There were other errors that should be corrected, including the earth curvature correction, the atmospheric refraction correction and the zero-point reading correction. The earth curvature correction is especially remarkable in this research when the distances between the control stations and the intersections are over kilometres.

The earth curvature correction, *δ_R_*, used to amend the surface curvature (earth radius *R*), causes errors in elevation, as shown in Fig. 1b. The longer the plane distance S is, the greater the error is. The height error over a distance of 2.5 km reaches half a metre. The atmospheric refraction correction, *δ_air_*, is mainly used to reduce the height error caused by vertical atmospheric refraction, which may be several centimetres or even over ten centimetres in our research. Both *δ_R_* and *δ_air_* can be calculated according to Eq. (6) [6].

(6) $$\begin{array}{c} \delta_{R}=\sqrt{S^{2}+R^{2}}-R\approx S^{2}/2R\\ \delta_{air}=kS^{2}/2R \end{array}\}$$

After observing each target and collecting its azimuthal record, the station was supposed to be aimed back again to check the horizontal azimuth, creating a zero-point reading. Due to instrument errors and in situ environmental impacts, the zero-point readings are usually not zero. In this research, half the zero-point readings were used for the horizontal azimuthal observations while the other half was used for the intersection's 2D error evaluations. On the other hand, the zero-point readings can be entirely assigned to the observations or be evenly divided into the 360°, but these two models are not recommended.

In this research, the coordinate reference systems is WGS84 and the value of *R* is 6,378,137 m. The vertical atmospheric refraction coefficient was fixed as k=0.1 according to the results of previous works in the polar regions [6]. Since an error of 0.01 in *k* would lead to a bias of ~0.002 m in the elevation calculation, the potential error in *k* can be ignored.

### Method validation

We have used this method to process the field azimuthal observations from Dalk Glacier, East Antarctica [1]. The two control stations were at two mountain peaks, and the targeted intersections were the stakes on the glacier. The stations’ positions were acquired from GPS measurements, and the stakes’ positions could be located with in situ azimuthal observations. After data processing using traditional space intersection method and our high-precision method, the comparison of position results showed that high-precision method corrected stake locations with a mean value of 0.156 m in height and 0.045 m in plane.

## References

[1] Ai S., Wang S., Li Y., Moholdt G., Zhou C., Liu L., Yang Y. (2019). High-precision ice-flow velocities from ground observations on Dalk Glacier, Antarctica. Polar Sci..

[2] Koch K.-.R. (1999). Parameter Estimation and Hypothesis Testing in Linear Models [M].

[3] Mikhail E.M., Gracie G. (1981). Analysis and Adjustment of Survey Measurements [M].

[4] Surveying adjustment discipline group in School of Geodesy and Geomatics of Wuhan University (2009). Error Theory and Foundation of Surveying adjustment [M].

[5] Surveying writing group of Wuhan Technical University of Surveying and Mapping (2000). Surveying.

[6] Wang Z., Ai S., Zhang S., Du Y. (2011). Elevation determination of nunataks in the Grove mountains. Adv. Polar Sci..

